# Analysis of Predictive Factors Associated with Unsuccessful Sentinel Lymph Node Mapping in Endometrial Carcinoma

**DOI:** 10.3390/cancers16213680

**Published:** 2024-10-31

**Authors:** Linas Andreika, Monika Šiaudinytė, Karolina Vankevičienė, Diana Ramašauskaitė, Vilius Rudaitis

**Affiliations:** 1 Clinic of Obstetrics and Gynecology, Institute of Clinical Medicine, Faculty of Medicine, Vilnius University, M. K. Čiurlionio Str. 21/27, LT-03101 Vilnius, Lithuania; diana.ramasauskaite@mf.vu.lt (D.R.); vilius.rudaitis@mf.vu.lt (V.R.); 2 Faculty of Medicine, Vilnius University, M. K. Čiurlionio Str. 21/27, LT-03101 Vilnius, Lithuania; monika.siaudinyte@mf.stud.vu.lt

**Keywords:** endometrial cancer, endometrial carcinoma, failed mapping, sentinel lymph node biopsy, sentinel lymph node mapping

## Abstract

Background: Sentinel lymph node (SLN) biopsy is recommended over systematic lymphadenectomy in early-stage endometrial cancer due to its lower morbidity and comparable detection rate. The objective of this study was to identify clinical factors associated with unsuccessful mapping. Methods: Between April 2020 and June 2024, 120 patients over the age of 18 and diagnosed with early-stage endometrial cancer were enrolled in this prospective study at a single institution. Demographic, clinicopathologic, and treatment data were collected and analyzed using descriptive statistics. Univariate and multiple linear regressions were performed to identify predictors of failed mapping. Results: The mean age of the patient cohort was 62.5 years (range 33 to 83), and the mean body mass index (BMI) was 32 kg/m^2^ (range 18 to 50). Patients underwent intracervical injections with methylene blue (MB), indocyanine green (ICG), or a combination of both tracers, with 40 patients in each group. A total of 108 patients (90.0%) were diagnosed with endometrioid carcinoma and 12 (10.0%) with non-endometrioid cancers. Additionally, 110 patients (91.7%) were diagnosed in early stages of the disease. The overall SLN detection rate was 73.4%, with bilateral detection at 49.2% and unilateral detection at 24.2%. Univariate analysis showed that older age (*p* < 0.001), menopause (*p* = 0.001), the use of MB as the sole tracer (*p* = 0.006), a shorter tumor-to-serosa distance (*p* = 0.048), and bulky lymph nodes (*p* = 0.18) were associated with unsuccessful mapping. Multiple linear regression model analysis identified age (*p* = 0.007), tracer type (*p* = 0.013), and enlarged lymph nodes (*p* = 0.013) as independent predictors of SLN mapping failure. Conclusions: Advanced age, tracer type, and intraoperative detection of enlarged lymph nodes were identified as independent risk factors for unsuccessful mapping in patients undergoing laparoscopic SLN mapping.

## Simple Summary

Sentinel lymph node (SLN) biopsy is recommended over systematic lymphadenectomy in early-stage endometrial cancer due to its lower morbidity and comparable detection rate. The objective of this study was to identify clinical factors associated with unsuccessful mapping. We conducted a prospective, single-institution study of 120 participants with early-stage uterine cancer. Univariate and multiple linear regressions were performed to identify predictors of unsuccessful mapping. Advanced age, tracer type, and intraoperative detection of enlarged lymph nodes were identified as independent risk factors for unsuccessful mapping in patients undergoing laparoscopic SLN mapping. These factors should be carefully considered during surgical planning and discussions prior to treatment. Additionally, the exploration of relationships between mapping failure and factors such as tumor distance to the serosa, uterine volume, and molecular tumor types, represents a promising area for future research that could complement our findings.

## 1. Introduction

Endometrial cancer ranks among the most common malignancies in developed countries. According to the World Cancer Research Fund (WCRF), there were 420,368 new cases of endometrial cancer worldwide in 2022, with projections suggesting an increase to over 600,000 cases by 2044 [1,2]. In Lithuania, uterine cancer ranks third among cancers in women and is predominantly diagnosed in postmenopausal individuals, with a median age of 62 years at diagnosis [3].

The majority of uterine cancers are of epithelial origin, with endometrioid adenocarcinomas being the most common subtype [4]. Surgical intervention, including total hysterectomy with bilateral salpingo-oophorectomy, is the primary treatment for early-stage endometrial cancer [5]. In endometrioid adenocarcinoma, lymph node metastasis is the most common pattern of extrauterine dissemination, and evaluating it is integral to surgical management. Lymph node status is a critical prognostic factor and a strong predictor of disease recurrence [6].

The optimal method for assessing lymph nodes has been a topic of debate. Historically, comprehensive lymphadenectomy, which involves dissection and evaluation of both pelvic and para-aortic nodes up to the renal veins, was standard practice for all patients. Recently, sentinel lymph node (SLN) mapping has gained prominence as a surgical technique due to its high accuracy in detecting metastases and its association with low morbidity, making it a preferred alternative to selective lymphadenectomy. This method has been increasingly utilized across all grades of endometrial cancer staging. To reduce adverse effects, the National Comprehensive Cancer Network (NCCN) and the European Society of Gynaecological Oncology (ESGO) now recommend a more selective approach to nodal evaluation using the sentinel lymph node algorithm. This is because the majority of patients undergoing systematic lymphadenectomy do not have metastases but do experience significant side effects from the procedure [7,8,9].

In order to perform SLN mapping, tracers such as blue dye, technetium-99m, indocyanine green (ICG), or combinations of thereof are used. ICG stands out among the various tracers used for SLN mapping due to its superior detection capabilities and clinical advantages. Among colorimetric dyes, such as methylene blue (MB), ICG provides the highest detection rates, significantly surpassing both MB and the former gold standard, the technetium-99m–MB combination [10]. ICG’s effectiveness is particularly notable in challenging cases, including obese patients, where it can be detected without tissue dissection, enhancing surgical outcomes [11].

Additionally, the use of near-infrared light for visualization is a critical advantage, as it remains invisible to the operator, thus preserving the operative field’s appearance and minimizing potential distractions. Overall, the reliability, safety, and time efficiency of ICG make it a preferred choice in SLN mapping, reinforcing its role as a valuable tool in modern surgical practice [10]. The procedure involves injecting the tracer into the cervix as a means to identify and biopsy endometrial cancer. The primary objective of SLN biopsy is to identify and remove the first lymph nodes that drain the primary tumor. If the SLN is cancer-free, it is inferred that the malignancy has not spread to other lymph nodes [12].

Given the advantages of SLN mapping over radical lymphadenectomy, it is important to note that bilateral SLN mapping fails in 20–25% of cases. Therefore, identifying predictive factors for SLN mapping failure is essential for optimizing surgical planning and minimizing the need for side-specific lymphadenectomies. Although various factors have been reported to influence SLN detection outcomes, comprehensive pooled data are still lacking. This study aimed to assess the predictive factors associated with failed SLN mapping in early-stage endometrial cancer patients undergoing SLN mapping via cervical injection of ICG, MB, or a combination of tracers [13,14,15].

## 2. Materials and Methods

### 2.1. Patient Population

A total of 120 patients, 18 years of age and older, with endometrial cancer (stage I, any grade, and histological type, according to the 2009 International Federation of Gynecology and Obstetrics (FIGO) classification) were enrolled in our institution’s prospective study between April 2020 and June 2024. Patients who did not provide written consent to participate, had allergies to MB dye or iodine, were unsuitable for curative surgery, had a history of bilateral pelvic or para-aortic lymphadenectomy, or were unsuitable for radiotherapy for other malignancies were excluded. In this study, patients were classified into risk groups according to the 2020 guidelines established by the European Society of Gynaecological Oncology (ESGO), the European Society for Radiotherapy and Oncology (ESTRO), and the European Society of Pathology (ESP) for the management of endometrial carcinoma [9].

Demographic, clinicopathologic, and treatment data were prospectively collected and recorded in a Microsoft Excel-based electronic database. Patient data included age, body mass index (BMI), menopausal status, number of pregnancies, and previous pelvic operations. Surgical data included the intracervical tracer technique used during the operation and lymph node staining. Pathologic data included stage, histology, molecular type, depth of invasion, presence of lymphovascular space invasion (LVSI), nodal status, and comorbidities such as myomatosis or adenomyosis, as well as uterine volume, tumor dimensions, and distance to the uterine serosa. The study was approved by the Vilnius Regional Biomedicine Research Ethics Committee (permit number 2020/3-1206-691 31 March 2020). All patients enrolled in the study provided written and informed consent.

### 2.2. SLN Mapping Procedure

In September 2019, a new protocol for the management and treatment of uterine cancer was introduced at the Gynecology Department of Vilnius University Hospital Santaros Clinics, approving SLN detection as an option for patients with initial-stage endometrial cancer undergoing laparoscopic total hysterectomy. In February 2020, fluorescent detection using ICG was initiated with the Olympus Visera Elite II OTV-S300 video system, the CH-S200-XZ-EB camera, ESG-400 and USG-400 generators, and a CLV-S200-IR light source (Olympus Corporation, Tokyo, Japan) for minimally invasive procedures.

All patients underwent staging with laparoscopic total hysterectomy, bilateral salpingo-oophorectomy, and SLN biopsy by one of the two surgeons dedicated to this trial. Enrolled patients were assigned to three study groups according to the tracers used: MB, ICG, and ICG–MB. The use of these three different mapping techniques—ICG, MB, and their combination (ICG + MB)—was part of a study protocol established in a previous publication, which aimed to compare the efficacy of these mapping methods. In this study continuation, the same approach was applied to further investigate predictive factors associated with unsuccessful sentinel lymph node mapping. Participants were randomized into study groups using a permuted block design and stratified randomization to reduce patient selection bias. Tracers were randomly assigned to the blocks, ensuring that the desired proportion of participants was evenly distributed across each block. In this trial, four blocks of 30 patients were established, maintaining a consistent number of participants per group. Within each block, 10 patients were allocated to the MB group, 10 to the ICG group, and 10 to the ICG–MB group. Stratification was applied based on measurable factors, like patient age, BMI, and tumor histology, that could influence study outcomes, ensuring no significant differences between the groups after randomization.

Cervical injections with tracers were performed after the induction of general anesthesia and before abdominal entry. The concentration of ICG in this study was 1.25 mg/mL. A 25 mg vial of ICG powder (Verdye, Diagnostic Green GmbH, Munich, Germany) was diluted with 20 mL of sterile 0.9% NaCl saline for each patient. A total of 8 mL of the ICG solution was injected into the cervix at the 2, 5, 7, and 10 o’clock positions. Of this, 1 mL was administered into the submucosal layer and 1 mL into the stroma, with a depth of penetration up to 1 cm. The MB solution, prepared by our hospital pharmacy, had a concentration of 0.25% (10 mg/mL). Using the same technique as for ICG, 8 mL of MB was injected into the cervix. In the combination group, both 8 mL of MB and 8 mL of ICG were injected at the same locations, with a 5 min interval between the two injections. Injections were performed by a surgeon or a resident doctor under the surgeon’s supervision. The Hohl uterine manipulator was not inserted until SLNs were detected intraoperatively.

Successful SLN mapping was defined as the identification of bilateral or aortic SLNs, whereas failed SLN mapping was defined as unilateral SLN detection or no SLN mapping at all. In cases where SLN detection was unsuccessful, patients in the low- and intermediate-risk groups did not undergo systematic lymphadenectomy unless lymph nodes appeared suspicious or were enlarged (>2 cm). For high-intermediate- and high-risk patients, a side-specific or comprehensive pelvic and aortic lymphadenectomy was performed if SLN mapping failed. However, due to comorbidities, some high-intermediate- and high-risk patients were not suitable for aortic lymphadenectomy, and only bilateral pelvic lymphadenectomy was performed. In accordance with ESGO guidelines, frozen sections were not performed during the operations.

### 2.3. Pathologic Evaluation

All SLNs were sent for pathologic evaluation separately from non-SLNs and were paraffin-embedded according to standard protocol. Hematoxylin and eosin (H&E) staining was performed. Ultrastaging was not conducted, as it is not routinely performed in our pathology center due to its high cost. Pathological disease staging was performed according to the 2009 FIGO classification after obtaining definitive histology results. Tumors were also classified using the standard molecular typing method from The Cancer Genome Atlas (TCGA), which categorizes samples into four groups: type 1—POLE ultra-mutation; type 2—microsatellite instability (MSI); type 3—low copy number (CN); and type 4—high CN. POLE ultra-mutated tumors were not detected in our study, as our pathology center did not have the capability to perform this test.

### 2.4. Data Analysis

Absolute and percentage frequencies were used to describe categorical variables, while mean or median values, standard deviations, and ranges were used to assess continuous variables. The normality of quantitative variable distributions was tested using the Shapiro–Wilk goodness-of-fit test. Comparisons between categorical variables were performed with the chi-square (χ2) test or Fisher’s exact test as needed. Comparisons between continuous variables were made using Student’s *t*-test for normally distributed data and the Mann–Whitney U test for non-normally distributed data. Univariate logistic regression was used to identify predictors of failed mapping. Multiple linear regression model analysis was conducted using all features that were statistically significant (*p* < 0.05) in the univariate analysis as independent variables, with bilateral mapping success or failure as the dependent variable. Covariates, such as age and menopause, were evaluated in separate multivariable models due to potential strong correlations among them. A *p* value of <0.05 was considered statistically significant. All analyses were conducted using IBM SPSS statistics 26.0 (IBM, Brøndby, Denmark). A post hoc power analysis was conducted using the G*Power software (latest version 3.1.9.7; Heinrich-Heine-Universität Düsseldorf, Düsseldorf, Germany). The significance level was set at α = 0.05, and the power level was 1 − β = 0.80. The required sample size for the study was found to be 120 patients.

## 3. Results

### 3.1. Patient Characteristics

During the study period, 120 patients met the inclusion criteria and were analyzed. The demographic characteristics and clinical–pathological data are detailed in Table 1. The mean age of the patients was 62.5 years, with a range from 33 to 83 years. In this group of patients, 85% were postmenopausal. The mean body mass index (BMI) was 32 kg/m^2^, ranging from 18 to 50 kg/m^2^. The vast majority of patients (88.8%) had a history of childbirth, with 9.4% having undergone a cesarean section. Approximately one-third (30.8%) of the cohort had a history of previous pelvic operations. MB, ICG, and ICG–MB combination intracervical injections were administered to 40 (33.33%) participants in each group, with no significant differences among the three patient groups. A total of 108 patients (90.0%) were diagnosed with endometrioid carcinoma, while 12 (10.0%) were diagnosed with non-endometrioid tumors. This included six cases of mixed carcinoma, five cases of serous carcinoma, and one case of clear-cell carcinoma. Among the patients, 107 (89.2%) had grade 1–2 tumors. Based on molecular typing, 32 patients (26.7%) were classified as molecular type 2 (MSI), 79 (65.8%) as type 3 (low CN), and 9 (7.5%) as type 4 (high CN). These findings are consistent with those reported by other authors [16]. The mean uterine volume was 121 mL, with a range from 12 to 858 mL. The mean maximum diameter of the tumor was 26.3 mm, with a range from 1 to 85 mm. The average tumor distance to the resection edge was 38.9 mm, with a range from 3 to 77 mm. The mean tumor distance to the uterine serosa was 7.7 mm, with a range from 1 to 53 mm. Seventy-eight patients (65.0%) had less than 50% myometrial infiltration. The majority (91.7%) of the patients were diagnosed with early-stage disease (76 cases (63.3%) were FIGO stage IA, 33 (27.5%) were stage IB, and 1 (0.8%) was stage II). The other 8.3% of patients were diagnosed with advanced disease: one patient (0.8%) with metastasis on the pelvic sidewall (stage IIIB) and nine patients (7.5%) with lymph node metastatic involvement (stage IIIC1). LVSI was found in 20 patients (16.7%). Bulky lymph nodes (2 cm in diameter or larger) were identified in 34.2% of the cohort. Among the 105 patients who had at least one lymph node removed, 9 patients (7.5%) had metastatic lymph node involvement. Histologically confirmed comorbidities included myomatosis, affecting 51.7% of the cohort, and adenomyosis, affecting 19.2% of the cohort.

### 3.2. SLN Mapping

Surgical and post-surgical details are outlined in Table 2. The overall detection rate, defined as the mapping of at least one SLN, was 73.4%. Among 59 patients (49.2%) with successful mapping, 56 (46.7%) had bilateral SLN mapping, and 3 (2.5%) had aortic SLNs. Unilateral SLN mapping was recorded in 29 cases (24.2%). Within the unilateral pelvic SLN mapping group, 14 patients (11.7%) had SLNs on the left hemipelvis, and 15 (12.5%) had them on the right. In the remaining 32 patients (26.6%), no SLN mapping was achieved. The bilateral mapping success rates were 27.5% for MB, 52.8% for ICG, and 67.5% for the ICG–MB combination, with a statistically significant difference (*p* = 0.006). Despite the apparent differences between the groups, statistical significance was only observed between the MB and ICG–MB cohorts (*p* = 0.001). No statistically significant differences were observed between the ICG and ICG–MB groups (*p* = 0.391). Notably, the ICG cohort demonstrated a trend toward significance compared to the MB group, although it was not reached (*p* = 0.056) (Table 3). A total of 13 patients (10.8%) underwent a unilateral pelvic, 31 (25.8%) a bilateral pelvic, and 12 (10.0%) a para-aortic lymphadenectomy due to unsuccessful mapping when the patient was either high-risk according to ESGO guidelines or enlarged lymph nodes were detected. Fifteen patients (12.5%) did not undergo pelvic or aortic lymph node dissection when SLN mapping failed due to low-risk tumors, intraoperative clinical conditions, morbid obesity, or poor overall health status. Complications were reported in eight (6.7%) patients: three cases of obturator nerve damage, four cases of postoperative infection, and one case of postoperative vaginal bleeding. There were no cases of allergic reactions to any of the tracers; however, three cases of brief saturation decrease associated with MB injection were recorded; these resolved without additional measures. No life-threatening complications or long-term damage was observed.

**Figure 1:**
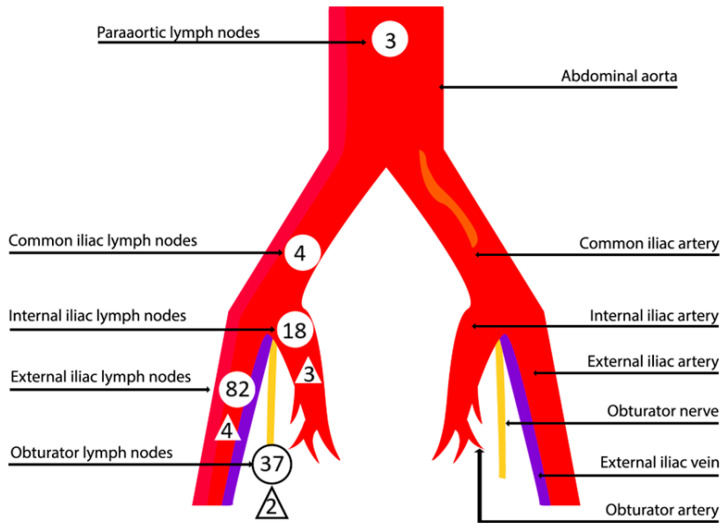
Sites for detecting SLNs (round shape) and metastases (triangular shape).

The details of SLNs are shown in Table 4 and Figure 1. A total of 144 SLN sites and 212 lymph nodes were detected, with the majority found in the external iliac region (56.9%). The median number of SLNs removed per patient was 2, with a range from 0 to 8. Out of the total SLNs recorded, 110 (51.9%) were in the external iliac region, 64 (30.2%) in the obturator region, 4 (1.9%) in the common iliac region, 29 (13.7%) in the internal iliac region, and 5 (2.3%) in the para-aortic region. After evaluating the final histology, 197 SLNs (92.9%) were negative for metastases, while 10 (4.7%) were positive. Empty nodes, defined as the absence of lymphatic tissue in presumed SLNs, were detected in five (2.4%) cases. Nodal involvement was observed in 9 (8.6%) patients out of 105 with known lymph node status who had at least one lymph node obtained: in 4 cases, patients had metastases in SLNs; in 3 cases, SLNs were not detected and metastases were found after performing full pelvic lymphadenectomy; and in 2 cases, patients had false-negative SLNs.

Univariate analysis (Table 1) found that older age, menopause, the use of MB as a sole tracer, a shorter tumor-to-serosa distance, and bulky lymph nodes were associated with failed mapping. Although patients with higher BMI, previous pelvic operations, and lower uterine volume showed tendencies toward failed mapping, statistical significance was not reached. Surprisingly, factors such as tumor grade, histological type, molecular type, tumor size, tumor distance to the resection edge, LVSI, lymph node involvement, and comorbidities like myomatosis or adenomyosis did not show tendencies toward unsuccessful mapping as initially presumed. Multiple linear regression model analysis revealed that age (*p* = 0.007), tracer type (*p* = 0.013), and enlarged lymph nodes (*p* = 0.013) were independent predictors of SLN mapping failure.

## 4. Discussion

Given the numerous benefits of sentinel lymph node biopsy compared to radical lymphadenectomy, understanding the factors that can negatively impact the successful mapping of sentinel lymph nodes is essential. Although previous studies have proposed potential predictors of unsuccessful sentinel lymph node mapping, variability in surgical techniques and the range of tracers used have complicated efforts to definitively determine the mechanisms contributing to mapping failure. Identifying risk factors for failed mapping is essential in clinical practice, as it impacts patient management and outcomes. While ICG is considered the optimal staining method, we have observed that a combined mapping technique can still provide significant benefits. Understanding these risk factors enables clinicians to inform patients about the likelihood of non-staining of the lymph nodes prior to surgery. In future research, we intend to develop prognostic models that incorporate criteria for potential failed mapping. These models will assist clinicians in evaluating the feasibility of complete lymphadenectomy while weighing the associated risks and benefits, ultimately leading to more personalized treatment strategies and improved patient outcomes.

Our study identified advanced age, the use of methylene blue as a sole tracer, and the presence of bulky lymph nodes as independent predictors of bilateral SLN mapping failure.

The overall SLN detection rate was 73.4%, the bilateral rate was 49.2%, and the unilateral rate was 24.2%. Comparatively, Sozzi et al. reported overall, bilateral, and unilateral detection rates of 96.3%, 76.3%, and 20.0%, respectively [17]. More recent retrospective analyses have reported enhanced bilateral SLN mapping success rates, ranging from approximately 74% to 78% [17,18]. Most of the authors used ICG as the sole tracer agent in their studies. An analysis of successful mapping rates stratified by tracer type in our study suggests that the reduced detection rates could be attributed to the lower efficacy of MB, with successful mapping rates of 27.5%, 52.8%, and 67.5% observed for MB, ICG, and the combination, respectively [19]. Numerous studies have shown that the use of ICG dye significantly improves SLN mapping success rates compared to the use of blue dye alone. However, MB has other advantages, such as cost-effectiveness and prolonged residence time in lymph nodes, compared to fast-spreading ICG, which stains too much of the lymphatic tissue. Moreover, MB is easy to obtain and does not require extra equipment, unlike ICG; therefore, it can be used for both open and minimally invasive surgeries. Because of its low cost and high accessibility, it is suitable for specialists in low-income countries, where minimally invasive surgery and high-cost tracers are unavailable. Despite showing lower detection rates compared to other tracers, it can still serve as a practical alternative to no SLN mapping at all [20]. On the other hand, our study showed that the best results can be achieved with the combination of MB and ICG. The same results were reflected in Holloway et al.’s study, thus keeping MB in the spotlight [21]. In our study, MB proved to be useful, particularly in cases where ICG stained the entire iliac region and SLNs were detected only by traces of MB. Additionally, several studies have analyzed not only the tracer itself but also the optimal dose of the tracer for SLN mapping. The results revealed that an ICG dose of less than 3 mL is associated with mapping failure [14,18]. Our study administered an accumulated dose of 8 mL (four sites, 2 mL per injection site) to all patients in accordance with our SLN biopsy protocol; therefore, we were unable to determine whether tracer dosages may have affected SLN detection rates.

Cianci et al. were the first to establish an independent correlation between advanced patient age and reduced SLN uptake, demonstrating a significant increase in unsuccessful mapping with each additional decade of age, using a cut-off of 65 years [22]. Similarly, our study revealed statistically significant differences, with patients experiencing unsuccessful mapping having a mean age of 66 years, compared to 58 years in those with successful mapping. Additionally, univariate analysis indicated a higher incidence of mapping failure in postmenopausal women compared to the premenopausal sample. However, multivariate analysis revealed that this factor is not independent, suggesting that the observed association may be attributable to advanced age rather than menopause itself. Similar findings are discussed in a meta-analysis by Raffone et al. It is hypothesized that the reduced SLN detection rate in older women may be linked to altered lymphatic flow due to increased vascular permeability associated with aging [23].

Obesity is another factor frequently associated with unsuccessful SLN mapping, although the literature presents conflicting findings. Ianieri et al. suggested that obesity is not a significant predictor of mapping failure, whereas Tanner et al. reported a reduced bilateral SLN detection rate in obese patients when using blue dye or ICG [15,24]. Vargiu et al. proved BMI was a significant predictor of unsuccessful bilateral SLN mapping, with a 1.156-fold increase in the risk of mapping failure for every five-unit increase in the BMI scale [25]. Our study found no significant connection between higher BMI and SLN mapping failure. This lack of significance may be attributed to the homogeneity of our cohort, where most patients were overweight, with a mean BMI of 32 kg/m^2^. When stratifying patients into two groups based on a BMI threshold of 30, we could see a tendency, although the differences remained statistically insignificant (*p* = 0.06). Clinically, surgeons often encounter challenges when operating on morbidly obese patients due to ventilation difficulties or the excessive amount of intra-abdominal adipose tissue, which can render SLN detection or dissection unfeasible. Only a small number of patients were excluded from our study because it was not possible to safely access the iliac zones.

The association between the intraoperative identification of bulky lymph nodes, defined as those exceeding 2 cm in diameter, and reduced success of SLN mapping has also been documented previously. As found by Garret et al., this phenomenon may be attributed to obstructed lymphatic flow and the presence of tumor thrombi, associated with high-volume lymphatic metastases; therefore, metastases in lymph nodes should also affect the mapping success rate [13,26]. Although elevated rates of bulky lymph nodes may be associated with macro- or micrometastases commonly observed in advanced disease stages, our analysis did not find a statistically significant relationship between mapping failure and any form of metastasis in any disease stage; this can most likely be attributed to a low number of patients with metastatic lymph nodes detected due to the absence of ultrastaging. For instance, Raffone et al.’s meta-analysis of six studies identified FIGO stages III and IV as independent predictive factors with *p* = 0.01 [18,23,24]. These findings suggest that preoperative imaging plays a critical role in identifying patients with bulky lymph nodes or metastatic disease who may be at an elevated risk of unsuccessful sentinel lymph node mapping [14]. Such an approach could significantly inform surgical planning.

We incorporated additional factors, such as the tumor distance to resection margins and tumor distance to the serosa, where their association with increased rates of unsuccessful SLN detection may also be attributed to disrupted lymphatic drainage pathways. While a shorter tumor-to-serosa distance was statistically significant in univariate analysis, this likely reflects advanced disease, where disrupted lymphatics impede SLN mapping. Proximity to the serosa may also correlate with LVSI, though we found no statistical significance between LVSI and mapping failure, possibly due to limited cases [17,23]. The SAGE study highlighted the association between LVSI and altered lymphatic drainage patterns as well; they identified LVSI as an independent predictor of unsuccessful SLN mapping [22]. Myometrial infiltration of more than 50% and a greater tumor diameter could also be associated with mapping failure due to similar mechanisms; however, neither our nor any other study data showed statistical significance [17,23].

Other factors potentially altering lymphatic drainage include increased uterine volume, which showed a near-significant association with mapping failure (*p* = 0.054). Although adenomyosis was not linked to mapping failure in our or other studies, the findings on fibroids are inconsistent [17,18]. Sozzi et al. observed a significant association with SLN failure in univariate analysis, while Tortorella et al. did not find any significance, even when categorizing fibroids by size. Additionally, while a higher number of prior births and cesarean deliveries were noted in the unsuccessful mapping group, neither reached statistical significance in our or any other studies [24,27].

Regarding the influence of histologic tumor subtype, studies comparing endometrioid and non-endometrioid types consistently indicate that the overall SLN detection rate is lower with an increased false-negative rate in women with non-endometrioid histology [14,27]. Interestingly, SLN mapping success did not significantly differ between tumor grades [13].

We classified uterine tumors into four molecular subtypes based on the TCGA method: POLE ultra-mutated, MSI, low CN, and high CN. The most influential subtypes affecting disease progression are POLE ultra-mutated and high CN [28]. However, due to limitations at our pathology center, POLE ultra-mutations were undetected and likely categorized as low CN. The remaining subtypes did not appear to influence mapping failure, likely due to cohort homogeneity. Consequently, our study may not accurately reflect the impact of molecular subtypes on mapping. Theoretically, high-CN tumors could correlate with failed mapping, while POLE ultra-mutated tumors might correlate with successful mapping (the same as with endometrioid and non-endometrioid tumors presented by other authors) [29]. A comparison with the existing literature is challenging due to limited data; however, we believe that the implementation of the FIGO 2023 uterine cancer classification will encourage authors to investigate this less studied factor [28].

Interestingly, previous pelvic surgeries appear to have no significant impact on SLN mapping success; however, the requirement for lysis of adhesions following prior surgeries is found to be significant, underscoring the need for careful management in patients with a history of previous surgeries or radiotherapy [17]. In these circumstances, mapping failure is likely attributable to an increased operative time prior to SLN identification and the presence of scar tissue in the pelvis from prior pelvic surgeries, particularly those involving the retroperitoneal space, which can impede lymphatic flow [18,30]. In such cases, delaying cervical dye injection until the completion of adhesion lysis may be a strategy worth considering.

The fact that our study demonstrated lower successful bilateral mapping rates compared to other studies emphasizes that theoretical detection rates may significantly decrease in a real-world environment. Although our study revealed intriguing tendencies, the limited sample size and cohort homogeneity affected the significance of the data, where clearly visible differences were found to be insignificant. As the size of the sample remains the main limitation of this study, we are actively continuing our work to enroll more patients and explore factors associated with failed mapping. The introduction of ultrastaging is one of our future goals, which could benefit patients and the study itself by possibly identifying more micrometastases. POLE ultra-mutated molecular subtype detection is in the early implementation stage within our pathology department, which will let us separate such low-risk patients from the low-CN group and help us avoid overtreatment. It should be noted, as mentioned earlier, that these findings are interim, and further research will contribute to a more comprehensive understanding of the subject. Moreover, it is essential to consider potential publication bias, where most of the studies are retrospective and only those with significant results are published, potentially distorting the overall evidence base in the field. Despite these limitations, our study demonstrates strengths, including new factors taken into consideration, appropriate research methodology, reliable data collection techniques, and statistical analysis that confirms previous findings.

## 5. Conclusions

Our study identified advanced age, tracer type, and bulky lymph nodes as independent predictors of bilateral sentinel lymph node mapping failure. These factors should be carefully considered in surgical planning and discussions. Additionally, exploring the relation between mapping failure and tumor distance to the serosa, uterine volume, and molecular types represents a promising area for future research that could complement our findings.

## Figures and Tables

**Table 1:** Univariate and multivariate analysis of bilateral sentinel lymph node mapping failure predictors.

Variable	Global Cohort (n = 120)	Successful Staining (n = 59)	Unsuccessful Staining (n = 61)	Univariate Analysis *p* Value	Multivariate Analysis *p* Value
Age (years) (mean (95% CI), range)	62.5 ± 9.7 (33 to 83)	58.4 ± 8.4 (33 to 75)	66.1 ± 9.4 (35 to 83)	**<0.001**	**0.007**
Menopause status				**0.001**	0.27
Yes	102 (85.0%)	41 (73.2%)	61 (95.3%)		
No	18 (15.0%)	15 (26.8%)	3 (4.7%)		
BMI (kg/m^2^) (mean (95% CI), range)	32.0 ± 6.0 (18 to 50)	30.4 ± 6.2 (18 to 50)	32.4 ± 5.6 (20 to 43)	0.13	
BMI ≥ 30 kg/m^2^				0.06	
Yes	75 (62.5%)	32 (54.2%)	43 (70.5%)		
No	45 (37.5%)	27 (45.8%)	18 (29.5%)		
Previous deliveries *				0.22	
Yes	103 (88.8%)	48 (85.7%)	55 (91.7%)		
No	13 (11.2%)	8 (14.3%)	5 (8.3%)		
Previous cesarean deliveries *				0.11	
Yes	10 (9.4%)	8 (14.3%)	2 (3.3%)		
No	106 (90.6%)	48 (85.7%)	58 (96.7%)		
Previous pelvic surgery				0.11	
Yes	37 (30.8%)	13 (23.2%)	24 (35.3%)		
No	83 (69.2%)	43 (76.8%)	40 (64.7%)		
Tracer agent used				**0.006**	**0.013**
MB	40 (33.3.3%)	11 (27.5%)	29 (72.5%)		
ICG	40 (33.3.3%)	21 (52.8%)	19 (47.2%)		
ICG + MB	40 (33.3.3%)	27 (67.5%)	13 (32.5%)		
Uterine volume (mL) (mean (95% CI), range)	121 ± 122 (12 to 858)	145.5 ± 155.1 (29 to 858)	100.5 ± 79.3 (12 to 392)	0.054	
Tumor max. diameter (mm) (mean (95% CI), range)	26.3 ± 17.1 (1 to 85)	24.1 ± 16.8 (1 to 70)	27.8 ± 17.5 (1 to 85)	0.27	
Tumor-to-resection-edge distance (mm) (mean (95% CI), range)	38.9 ± 16.9 (3 to 77)	40.9 ± 19.1 (3 to 77)	37.3 ± 15.0 (4 to 60)	0.38	
Tumor-to-serosa distance (mm) (mean (95% CI), range)	7.7 ± 8.2 (1 to 53)	9.8 ± 10.5 (1 to 53)	5.4 ± 3.9 (1 to 15)	**0.048**	0.87
Tumor histological type				0.71	
endometrioid	108 (90.0%)	51 (91.1%)	57 (89.1%)		
non-endometrioid	12 (10.0%)	5 (8.9%)	7 (10.9%)		
Tumor grading				0.82	
G1	90 (75.0%)	43 (76.8%)	47 (73.4%)		
G2	17 (14.2%)	8 (14.3%)	9 (14.1%)		
G3	13 (10.8%)	5 (8.9%)	8 (12.5%)		
Molecular type				0.68	
Type 2	32 (26.7%)	16 (28.6%)	16 (25.0%)		
Type 3	79 (65.8%)	37 (66.1%)	42 (65.6%)		
Type 4	9 (7.5%)	3 (5.4%)	6 (9.4%)		
Myometrial infiltration (50% and more)				0.17	
Yes	42 (35.0%)	16 (17.9%)	26 (15.6%)		
No	78 (65.0%)	40 (82.1%)	38 (84.4%)		
FIGO stage				0.66	
Early (I and II)	110 (91.7%)	52 (92.9%)	58 (90.6%)		
Advanced (III and IV)	10 (8.3%)	4 (7.1%)	6 (9.4%)		
LVSI				0.74	
Yes	20 (16.7%)	10 (17.9%)	10 (15.6%)		
No	100 (83.3%)	46 (82.1%)	54 (84.4%)		
Lymph nodes >2 cm				**0.018**	**0.013**
Yes	41 (34.2%)	13 (23.2%)	28 (43.8%)		
No	79 (65.8%)	43 (76.8%)	36 (56.3%)		
Lymph node involvement **				0.21	
Yes	9 (7.5%)	3 (5.4%)	6 (9.4%)		
No	96 (92.5%)	53 (94.6%)	43 (90.6%)		
Myomatosis				0.45	
Yes	62 (51.7%)	31 (55.4%)	31 (48.4%)		
No	58 (48.3%)	25 (44.6%)	33 (51.6%)		
Adenomyosis				0.9	
Yes	23 (19.2%)	11 (19.6%)	12 (18.8%)		
No	97 (80.8%)	45 (80.4%)	52 (81.2%)		

Abbreviations: BMI—body mass index; max.—maximum; FIGO—International Federation of Gynecology and Obstetrics; LVSI—lymphovascular space invasion; *—missing data of 4 patients; **—15 patients did not undergo any type of lymphadenectomy.

**Table 2:** Surgical and post-surgical details of sentinel lymph nodes.

Variables	N (%)
Sentinel node detection	
Absence	32 (26.6)
Unilateral pelvic	29 (24.2)
Left	14 (11.7)
Right	15 (12.5)
Bilateral pelvic	56 (46.7)
Aortic	3 (2.5)
Total successful	59 (49.2)
Overall detection rate	88 (73.4)
Pelvic lymphadenectomy	
Not performed	76 (63.4)
Unilateral	13 (10.8)
Bilateral	31 (25.8)
Para-aortic lymphadenectomy	
Not performed	108 (90)
Performed	12 (10)
Complications	
Yes	8 (6.7)
No	112 (93.3)

**Table 3:** Tracer successful mapping rates.

Variable	Global Cohort	MB (a)	ICG (b)	ICG–MB (c)	*p* Value	a to b	b to c	a to c
Successful mapping (n (%))	59 (49.2)	11 (27.5)	21 (52.8)	27 (67.5)	0.006	0.056	0.391	0.001

Abbreviations: a—methylene blue group; b—indocyanine green group; c—indocyanine green and methylene blue combination group.

**Table 4:** Sentinel lymph node biopsy details.

Variable	N
Total number of sentinel nodes removed	212
Median number of sentinel nodes removed (range)	2 (0–8)
Total sentinel node sites	144
External iliac	82 (56.9%)
Obturator	37 (25.7%)
Internal iliac	18 (12.5%)
Common iliac	4 (2.8%)
Para-aortic	3 (2.1%)
Sentinel node number in sites	
External iliac	110 (51.9%)
Obturator	64 (30.2%)
Internal iliac	29 (13.7%)
Common iliac	4 (1.9%)
Para-aortic	5 (2.3%)
Histological state of sentinel nodes	
Negative	197 (92.9%)
	10 (4.7%)
Positive	5 (2.4%)
Empty nodes	

## Data Availability

Data are currently unavailable due to privacy restrictions, as the research is still ongoing. The data will be shared after the study is completed and the final results are published.

## References

[1] Endometrial Cancer Statistics|World Cancer Research Fund International. WCRF International. https://www.wcrf.org/cancer-trends/endometrial-cancer-statistics/.

[2] Yang L., Yuan Y., Zhu R., Zhang X. (2023). Time trend of global uterine cancer burden: An age-period-cohort analysis from 1990 to 2019 and predictions in a 25-year period. BMC Women’s Health.

[3] WHO (2024). Global Cancer Observatory: Cancer Today. https://gco.iarc.who.int/today/.

[4] Rabban J.T., Gilks C.B., Malpica A., Matias-Guiu X., Mittal K., Mutter G.L., Oliva E., Parkash V., Ronnett B.M., Staats P. (2019). Issues in the Differential Diagnosis of Uterine Low-grade Endometrioid Carcinoma, Including Mixed Endometrial Carcinomas: Recommendations from the International Society of Gynecological Pathologists. Int. J. Gynecol. Pathol..

[5] Morrison J., Balega J., Buckley L., Clamp A., Crosbie E., Drew Y., Durrant L., Forrest J., Fotopoulou C., Gajjar K. (2022). British Gynaecological Cancer Society (BGCS) uterine cancer guidelines: Recommendations for practice. Eur. J. Obstet. Gynecol. Reprod. Biol..

[6] Kalampokas E., Giannis G., Kalampokas T., Papathanasiou A.-A., Mitsopoulou D., Tsironi E., Triantafyllidou O., Gurumurthy M., Parkin D.E., Cairns M. (2022). Current Approaches to the Management of Patients with Endometrial Cancer. Cancers.

[7] Abu-Rustum N.R., Yashar C.M., Arend R., Barber E., Bradley K., Brooks R., Campos S.M., Chino J., Chon H.S., Crispens M.A. (2024). NCCN Guidelines Index Table of Contents Discussion.

[8] Suchetha S., Mathew A.P., Rema P., Thomas S. (2021). Pattern of Lymph Node Metastasis in Endometrial Cancer: A Single Institution Experience. Indian J. Surg. Oncol..

[9] ESGO/ESTRO/ESP Guidelines for the Management of Patients with Endometrial Carcinoma|International Journal of Gynecologic Cancer. https://ijgc.bmj.com/content/31/1/12.

[10] Buda A., Crivellaro C., Elisei F., Di Martino G., Guerra L., De Ponti E., Cuzzocrea M., Giuliani D., Sina F., Magni S. (2016). Impact of Indocyanine Green for Sentinel Lymph Node Mapping in Early Stage Endometrial and Cervical Cancer: Comparison with Conventional Radiotracer 99mTc and/or Blue Dye. Ann. Surg. Oncol..

[11] Eriksson A.G.Z., Montovano M., Beavis A., Soslow R.A., Zhou Q., Abu-Rustum N.R., Gardner G.J., Zivanovic O., Barakat R.R., Brown C.L. (2016). Impact of Obesity on Sentinel Lymph Node Mapping in Patients with Newly Diagnosed Uterine Cancer Undergoing Robotic Surgery. Ann. Surg. Oncol..

[12] Nieweg O.E., Uren R.F., Thompson J.F. (2015). The History of sentinel lymph node biopsy. Cancer J..

[13] Garrett A.A., Wield A., Mumford B., Janmey I., Wang L., Grosse P., MacArthur E., Buckanovich R., Courtney-Brooks M., Sukumvanich P. (2022). Clinical factors associated with failed sentinel lymph node mapping in endometrial cancer. Gynecol. Oncol. Rep..

[14] Taşkın S., Sarı M.E., Altın D., Ersöz C.C., Gökçe A., Yüksel S., Kankaya D., Ortaç F. (2019). Risk factors for failure of sentinel lymph node mapping using indocyanine green/near-infrared fluorescent imaging in endometrial cancer. Arch. Gynecol. Obstet..

[15] Tanner E.J., Sinno A.K., Stone R.L., Levinson K.L., Long K.C., Fader A.N. (2015). Factors associated with successful bilateral sentinel lymph node mapping in endometrial cancer. Gynecol. Oncol..

[16] Alexa M., Hasenburg A., Battista M.J. (2021). The TCGA Molecular Classification of Endometrial Cancer and Its Possible Impact on Adjuvant Treatment Decisions. Cancers.

[17] Sozzi G., Fanfani F., Berretta R., Capozzi V.A., Uccella S., Buono N., Giallombardo V., Di Donna M.C., Monterossi G., Restaino S. (2020). Laparoscopic sentinel node mapping with intracervical indocyanine green injection for endometrial cancer: The SENTIFAIL study—A multicentric analysis of predictors of failed mapping. Int. J. Gynecol. Cancer.

[18] Tortorella L., Casarin J., Multinu F., Cappuccio S., McGree M.E., Weaver A.L., Langstraat C.L., Keeney G.L., Kumar A., Melis G.B. (2019). Sentinel lymph node biopsy with cervical injection of indocyanine green in apparent early-stage endometrial cancer: Predictors of unsuccessful mapping. Gynecol. Oncol..

[19] Andreika L., Vankevičienė K., Ramašauskaitė D., Rudaitis V. (2024). Visualization Methods for Uterine Sentinel Lymph Nodes in Early-Stage Endometrial Carcinoma: A Comparative Analysis. Diagnostics.

[20] Bistas E., Sanghavi D.K. (2024). Methylene Blue.

[21] Holloway R.W., Ahmad S., Kendrick J.E., Bigsby G.E., Brudie L.A., Ghurani G.B., Stavitzski N.M., Gise J.L., Ingersoll S.B., Pepe J.W. (2017). A Prospective Cohort Study Comparing Colorimetric and Fluorescent Imaging for Sentinel Lymph Node Mapping in Endometrial Cancer. Ann. Surg. Oncol..

[22] Cianci S., Rosati A., Vargiu V., Capozzi V.A., Sozzi G., Gioè A., Alletti S.G., Ercoli A., Cosentino F., Berretta R. (2021). Sentinel Lymph Node in Aged Endometrial Cancer Patients “The SAGE Study”: A Multicenter Experience. Front. Oncol..

[23] Raffone A., Fanfani F., Raimondo D., Rovero G., Renzulli F., Travaglino A., De Laurentiis U., Santoro A., Zannoni G.F., Casadio P. (2023). Predictive factors of sentinel lymph node failed mapping in endometrial carcinoma patients: A systematic review and meta-analysis. Int. J. Gynecol. Cancer.

[24] Ianieri M.M., Puppo A., Novelli A., Campolo F., Staniscia T., Di Martino G., Piovano E., Bruni F., Roviglione G., Mautone D. (2019). Sentinel Lymph Node Biopsy in the Treatment of Endometrial Cancer: Why We Fail? Results of a Prospective Multicenter Study on the Factors Associated with Failure of Node Mapping with Indocyanine Green. Gynecol. Obstet. Investig..

[25] Vargiu V., Rosati A., Capozzi V.A., Sozzi G., Gioè A., Berretta R., Chiantera V., Scambia G., Fanfani F., Cosentino F. (2022). Impact of Obesity on Sentinel Lymph Node Mapping in Patients with apparent Early-Stage Endometrial Cancer: The ObeLyX study. Gynecol. Oncol..

[26] Goyal A., Douglas-Jones A., Newcombe R., Mansel R., ALMANAC Trialists Group (2004). Predictors of non-sentinel lymph node metastasis in breast cancer patients. Eur. J. Cancer.

[27] Body N., Grégoire J., Renaud M.-C., Sebastianelli A., Grondin K., Plante M. (2018). Tips and tricks to improve sentinel lymph node mapping with Indocyanin green in endometrial cancer. Gynecol. Oncol..

[28] Zheng W. (2023). Molecular Classification of Endometrial Cancer and the 2023 FIGO Staging: Exploring the Challenges and Opportunities for Pathologists. Cancers.

[29] Jumaah A.S., Al-Haddad H.S., McAllister K.A., Yasseen A.A. (2022). The clinicopathology and survival characteristics of patients with POLE proofreading mutations in endometrial carcinoma: A systematic review and meta-analysis. PLoS ONE.

[30] Khoury-Collado F., Glaser G.E., Zivanovic O., Sonoda Y., Levine D.A., Chi D.S., Gemignani M.L., Barakat R.R., Abu-Rustum N.R. (2009). Improving sentinel lymph node detection rates in endometrial cancer: How many cases are needed?. Gynecol. Oncol..

